# First report of *Rhinocladiella mackenziei* osteomyelitis with CNS dissemination in a pediatric CGD patient post–HSCT: A case report and literature review

**DOI:** 10.1016/j.mmcr.2026.100814

**Published:** 2026-08-03

**Authors:** Tasneem Shamsi Basha, Hari Pankaj Vanam, Akela Ghazawi, Mansi Sachdev, Shazia Chiken, Rubina Monga, Fatima Al Dhaheri

**Affiliations:** a Sheikh Khalifa Medical City, Abu Dhabi, United Arab Emirates; b College of Medicine and Health Sciences, Al Ain, Abu Dhabi, United Arab Emirates; c Abu Dhabi Stem Cell Center, Abu Dhabi, United Arab Emirates; d Al Jalila Children's Hospital, Dubai, United Arab Emirates; e Dubai Health Authority, Dubai, United Arab Emirates

**Keywords:** *Rhinocladiella mackenziei*, Cerebral phaeohyphomycosis, Chronic granulomatous disease, Hematopoietic stem cell transplantation, Brain abscess, Invasive fungal infection, pediatric patient, Dematiaceous fungi

## Abstract

**Background:**

*Rhinocladiella mackenziei* is a rare neurotropic dematiaceous fungus that causes cerebral phaeohyphomycosis, predominantly in immunocompetent adults with a geographical predilection to the Middle East. Pediatric infection is exceptionally rare, and to our knowledge, no cases of combined osseous and central nervous system involvement following hematopoietic stem cell transplantation (HSCT) have been reported.

**Case:**

We report a male child with autosomal recessive chronic granulomatous disease (CGD) who developed traumatic forearm osteomyelitis due to *R. mackenziei* several months after haploidentical HSCT. Subsequent neuroimaging demonstrated intracerebral abscesses despite immune reconstitution. The infection persisted despite prolonged combination antifungal therapy with voriconazole and amphotericin B and ultimately required neurosurgical excision. The patient recovered without neurological sequelae and continues on long-term posaconazole therapy.

**Conclusion:**

This case highlights *R. mackenziei* as a cause of severe, invasive, disseminated fungal disease in pediatric post-bone marrow transplant patients and demonstrates extracerebral inoculation with subsequent CNS dissemination. It also underscores the importance of early recognition and a combined medical-surgical approach in management of refractory disease.

## 1 Introduction

Dematiaceous (melanized) fungi are an uncommon but increasingly recognized cause of invasive disease, particularly in immunocompromised hosts [1,2]. Among these, *Rhinocladiella mackenziei* is a rare, highly neurotropic mold most often presenting as cerebral abscesses in adults and is associated with a high mortality rate approaching 70-100% despite combined surgical and antifungal therapy [[2], [3], [4], [5], [6]].

Despite growing recognition, clinical experience with *R. mackenziei* cerebral phaeohyphomycosis in pediatric populations remains exceedingly limited [3,6]. To our knowledge, no pediatric cases of disseminated *R. mackenziei* infection with combined osseous and central nervous system involvement following hematopoietic stem cell transplantation (HSCT) have been described.

We report a pediatric case of invasive *Rhinocladiella mackenziei* infection presenting as osteomyelitis with secondary cerebral abscess formation following haploidentical allogeneic HSCT. This case illustrates the organism's neurotropism, the diagnostic and therapeutic challenges encountered, and the importance of early imaging, timely microbiologic and molecular diagnosis, and combined medical–surgical management in achieving a favorable outcome.

## 2 Case report

A male child with a history of recurrent infections during the first three months of life, including repeated cervical abscesses requiring surgical drainage and episodic febrile illnesses, was diagnosed with autosomal recessive chronic granulomatous disease (CGD) at the age of 3 months following immunologic and genetic evaluation, which identified a homozygous pathogenic variant in NCF1 (c.579G > A, p.Trp193Ter). Baseline neuroimaging at the time of diagnosis was reportedly unremarkable.

Despite antimicrobial prophylaxis and supportive care, he continued to experience significant infectious complications, and at 4 years of age he underwent haploidentical allogeneic HSCT from a sibling donor following reduced-toxicity conditioning. The early post-transplant course was complicated by cytomegalovirus reactivation which was treated with antiviral therapy and followed by maintenance antiviral prophylaxis. Neutrophil and platelet engraftment were achieved, and subsequent immune reconstitution was demonstrated by donor chimerism exceeding 95% across cell lineages and normalization of oxidative burst testing. Initial post-transplant immunosuppression consisted of tacrolimus, mycophenolate mofetil for graft-versus- host disease (GVHD) prophylaxis, together with standard antimicrobial prophylaxis including valganciclovir and voriconazole. The early post-transplant course was complicated by acute cutaneous GVHD characterized by a non-pruritic maculopapular rash involving the extremities, confirmed histologically on skin biopsy, and associated with acute kidney injury and electrolyte disturbances. This was managed with systemic corticosteroids (prednisolone 1 mg/kg/day for two weeks)and temporary reduction of calcineurin inhibitor dosing, with subsequent clinical resolution of cutaneous manifestations. Steroids were gradually tapered over approximately four weeks and discontinued.

At approximately Day 150 following haploidentical allogeneic HSCT and shortly after the period of augmented immunosuppression for GVHD, the child presented with localized right forearm pain, swelling, and functional limitation after minor trauma.

**Fig. 1:**
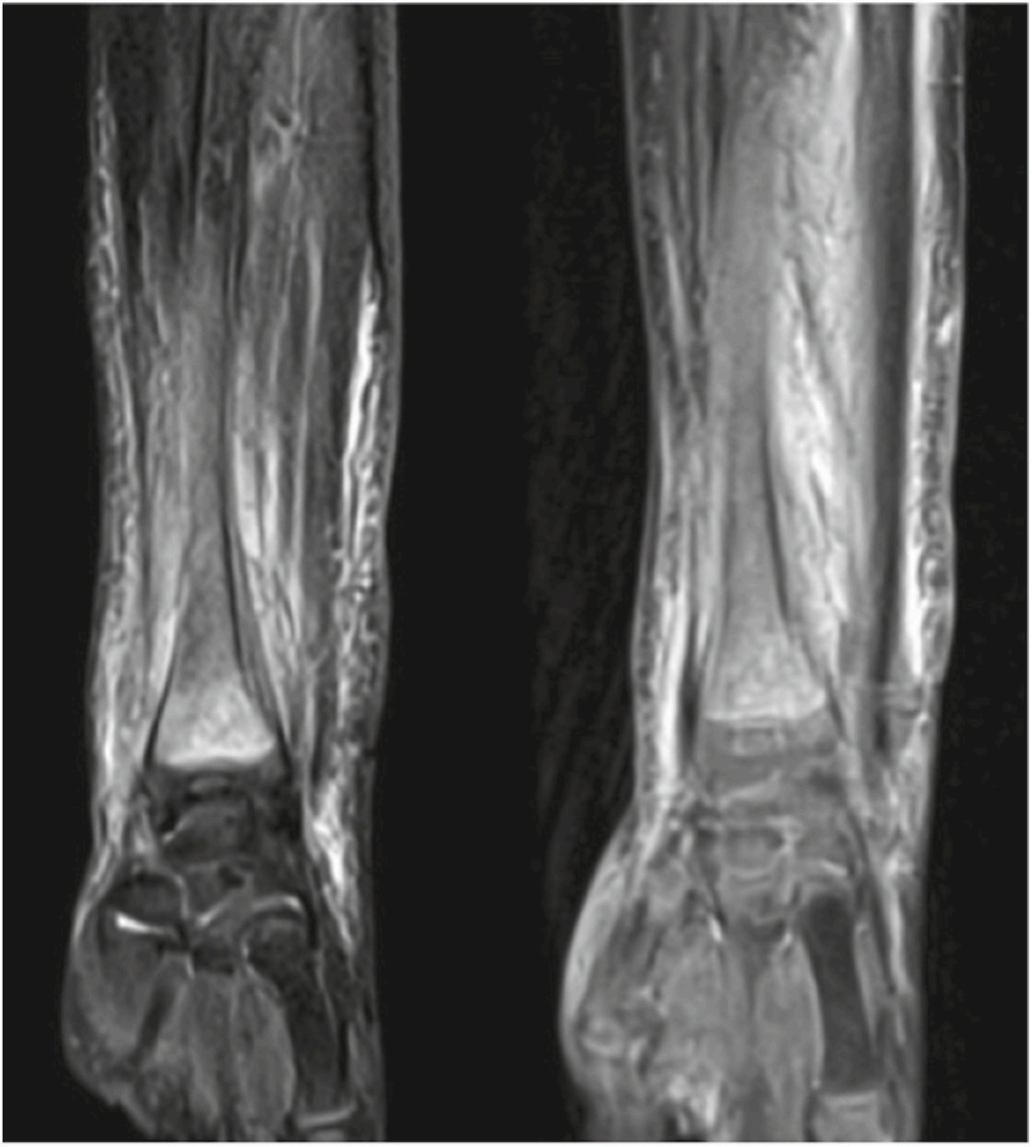
MRI of the Right forearm demonstrating cortical irregularity of the distal radius and ulna with surrounding soft tissue edema consistent with osteomyelitis.

On Day 0 of onset of symptoms, imaging demonstrated features consistent with osteomyelitis (Fig. 1), empiric antimicrobial therapy was initiated with ceftriaxone 75 mg/kg/day and clindamycin 40 mg/kg/day, surgical debridement was performed at Day 2. Due to rising inflammatory markers, the regimen was switched to piperacillin-tazobactam 300 mg/kg/day and vancomycin 60 mg/kg/day, which was later escalated to linezolid 30 mg/kg/day.

Routine bacterial cultures were negative. The patient received intravenous antibiotics for 20 days and was discharged on day 20 with a one-month course of oral ciprofloxacin 30 mg/kg/day; linezolid was withheld at discharge due to neutropenia. Following discharge, and on Day 39 from initial presentation, fungal culture of the bone specimen obtained at the time of debridement yielded a melanized (dematiaceous) mold morphologically suggestive of a *Rhinocladiella*-like species, prompting the initiation of oral voriconazole 9 mg/kg/dose twice daily. Notably, direct potassium hydroxide (KOH) mount of this specimen was initially negative for fungal elements. Fungal growth became evident after ∼2 weeks of incubation, with identifiable morphological features and condition patterns consistent with slow-growing dematiaceous fungi. In light of the delayed culture positivity, additional laboratory evaluation was undertaken to exclude contamination. A second specimen was processed from retained material originally submitted for mycobacterial culture. Repeat cultures of both specimens were performed and yielded the same mold with consistent morphology. Furthermore, a retrospective review of histopathological (HP) examination results revealed acute suppurative inflammation consistent with abscess formation, with rare fungal elements, including spores and hyphae-like structures, identified within the inflammatory background (Fig. 4A and B). Gomori methenamine silver (GMS) staining was equivocal, likely reflecting the low fungal burden in tissue, and no evidence of malignancy was observed. The concordance between culture findings and the retrospective histopathological detection of fungal elements confirmed the clinical significance of the isolate.

**Fig. 2:**
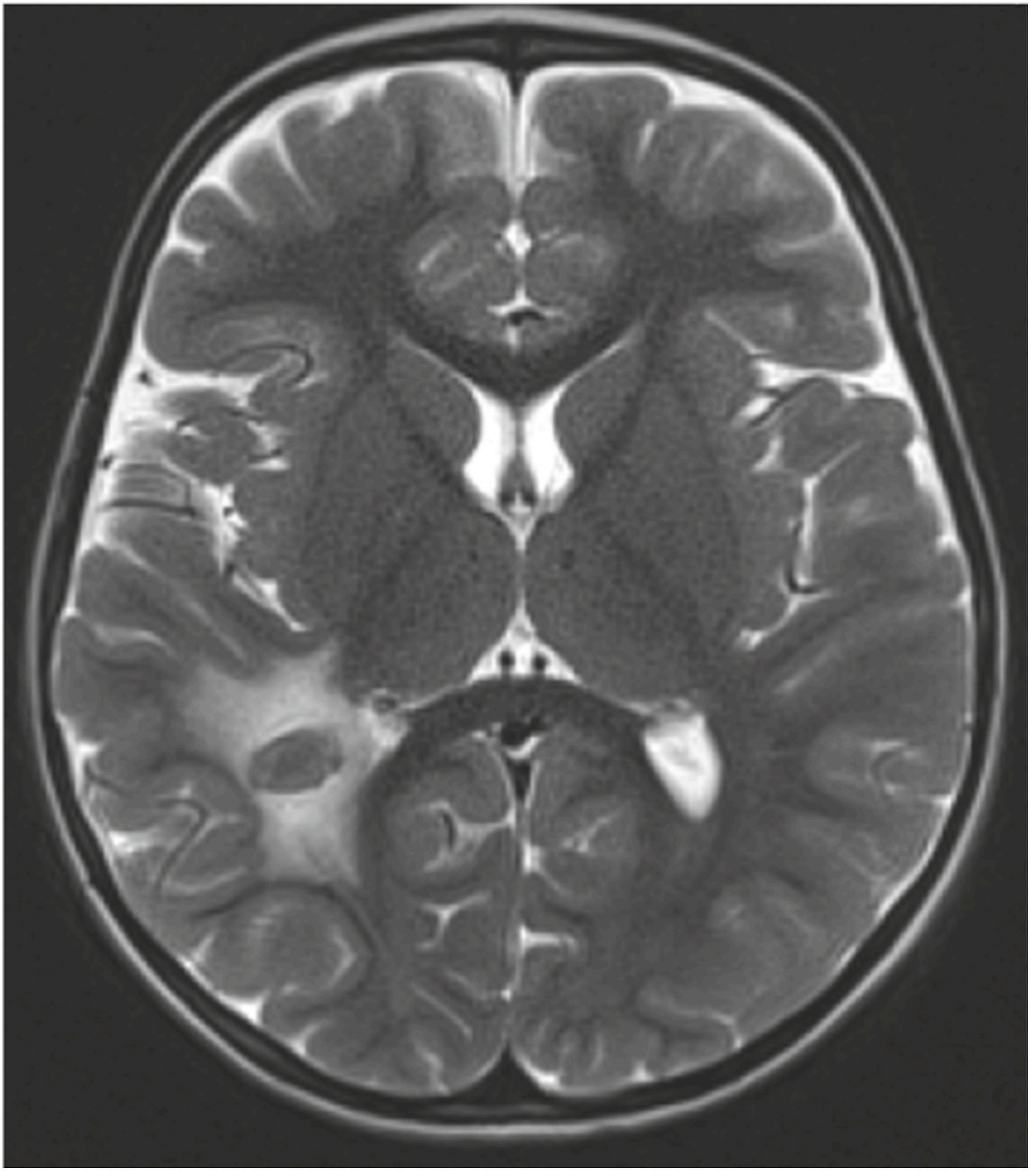
Post-contrast axial MRI showing a peripherally enhancing lesion in the right temporoparietal region with restricted diffusion and prominent vasogenic edema.

Furthermore, MALDI-TOF mass spectrometry (VITEK MS) failed to generate a reliable identification or matching spectrum. Given this limitation, definitive species identification was established at the United Arab Emirates University Fungal Reference Laboratory (UAEU-FRL) using a combination of culture morphology and molecular sequencing, providing confirmation of the organism as the true etiological agent. To evaluate for disseminated disease, whole-body imaging with positron emission tomography computed tomography (PET-CT) was requested; however, this could not be performed. On Day 72, the patient developed low-grade fever and diarrhoea while receiving oral voriconazole for 33 days, prompting hospital admission. He remained clinically stable and exhibited no focal neurological deficits. Contrast-enhanced magnetic resonance imaging (MRI) of the brain was therefore obtained to screen for CNS involvement, given the organism's known neurotropism, and revealed a circumscribed ring-enhancing lesion at the junction of the right temporal and parietal lobes measuring (1.9 x 1.7 × 1.4 cm) with diffusion restriction and marked surrounding vasogenic edema, consistent with an intracerebral abscess (Fig. 2). A second smaller enhancing focus (4 mm) was present in the inferior right-frontal lobe.

**Fig. 3:**
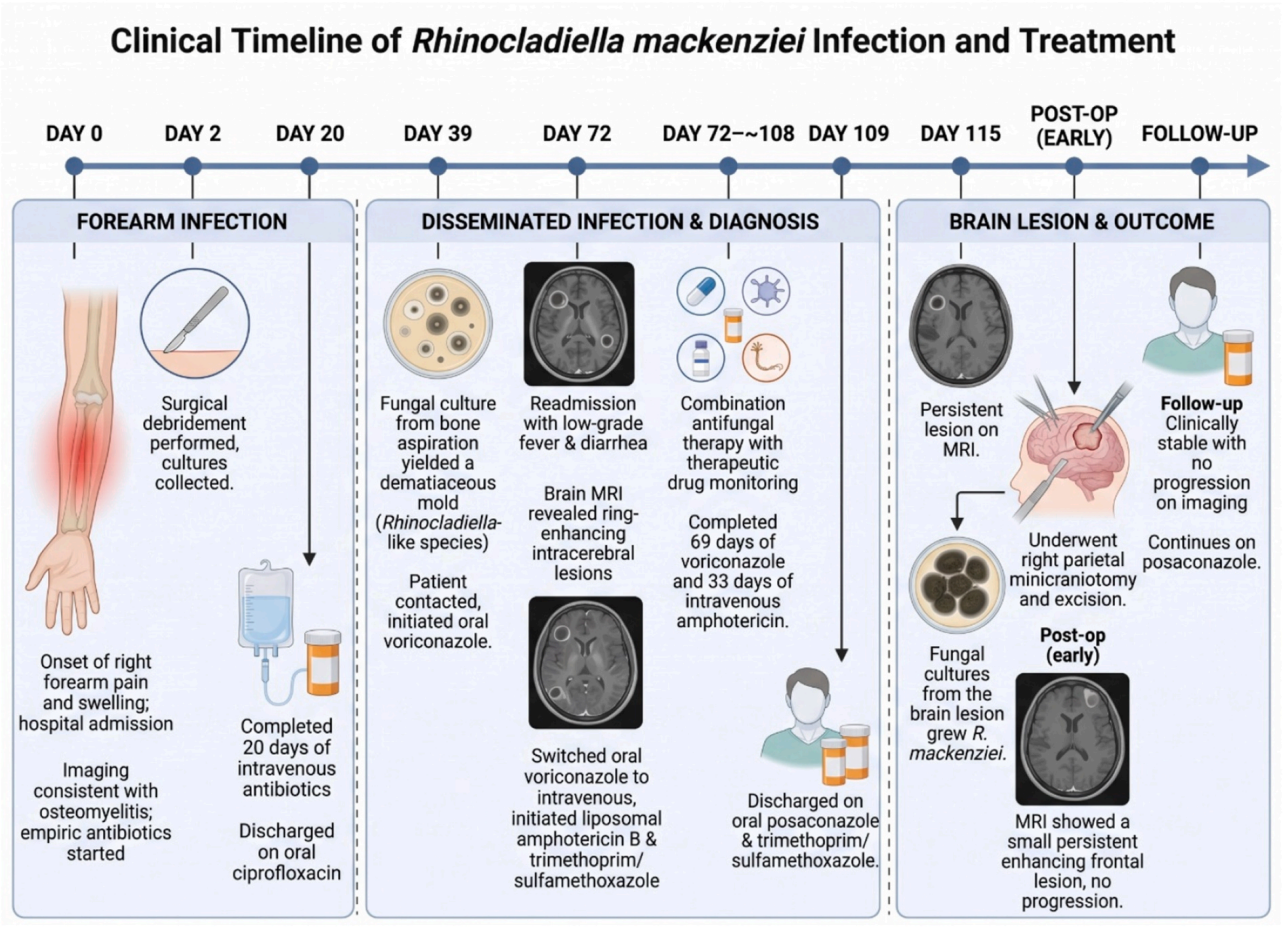
Schematic clinical timeline depicting progression of *Rhinocladiella mackenziei* infection from initial forearm involvement to disseminated central nervous system disease with corresponding diagnostic and therapeutic interventions.

Voriconazole was transitioned from oral to intravenous administration and intravenous liposomal amphotericin B was initiated concurrently at a dose of 70 mg once daily. Voriconazole therapeutic drug monitoring was performed during intravenous therapy; trough levels remained persistently subtherapeutic (0.18 mg/L and 0.6 mg/L) despite sequential dose escalation from 9 mg/kg/dose twice daily to 10 mg/kg/dose twice daily and subsequently 11 mg/kg twice daily. Total voriconazole exposure was 69 days (33 days oral, 36 days intravenous), with liposomal amphotericin B administered for 33 days. Since voriconazole levels remained subtherapuetic despite dose escalation, the patient was switched to intravenous posaconazole 600 mg daily to achieve better absorption. A detailed timeline of antifungal therapy is provided in Fig. 3.

**Fig. 4:**
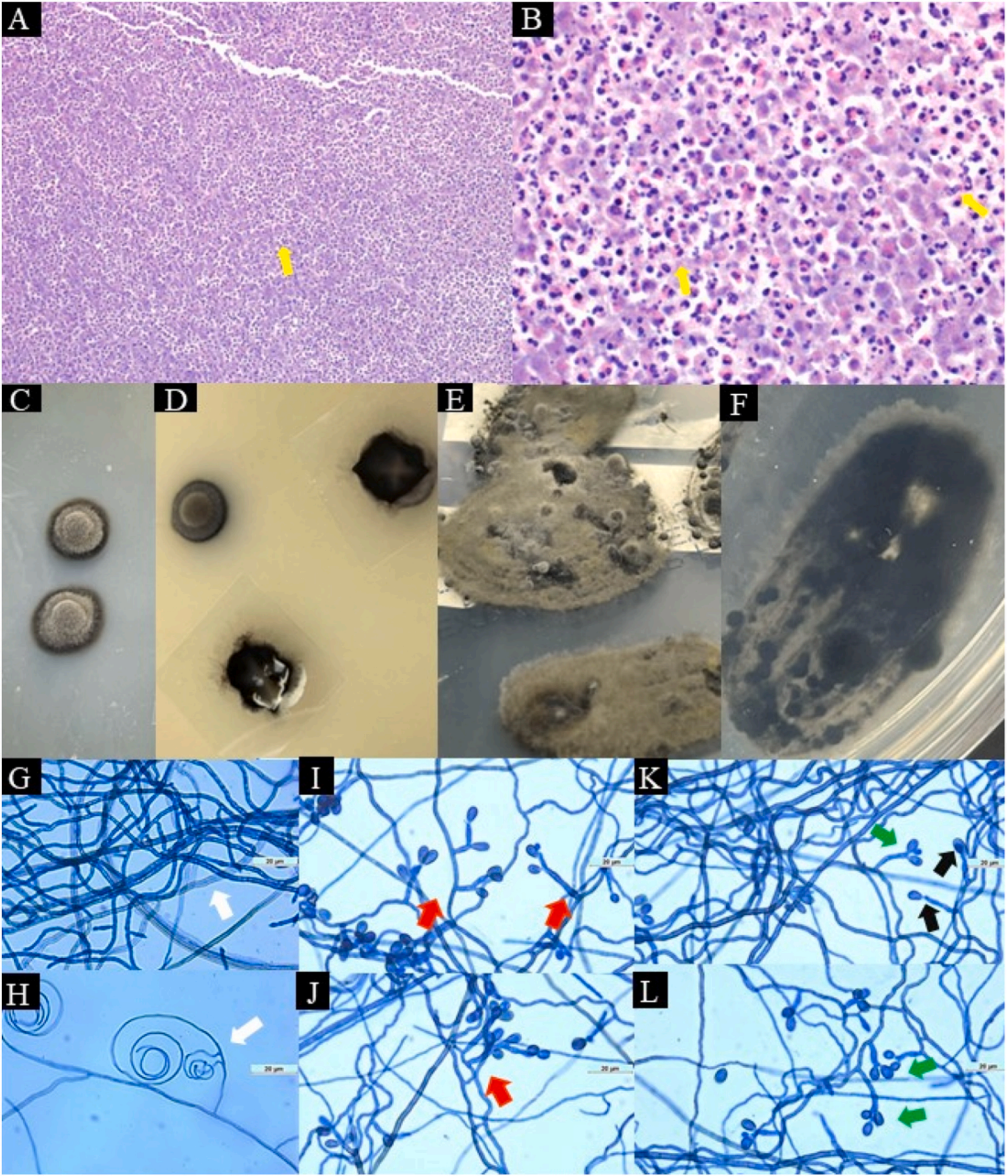
**Histopathological (HP) and Mycological Features of *Rhinocladiella mackenziei*.** (A–B) HP findings from bone biopsy of the right radius. Acute suppurative inflammation consistent with abscess formation, with dense inflammatory infiltrates and rare putative fungal-like structures, including spores (yellow arrows) (Hematoxylin and Eosin stain). **(C–F)** Colony morphology following incubation at 28–30 °C. The isolate demonstrated slow growth, with initial visible colonies after 5–7 days and full morphological development by 10–14 days. Colonies were olivaceous-black to black with a velvety texture and darkly pigmented reverse, characteristic of dematiaceous fungi. **(C)** Obverse on Potato Dextrose Agar (PDA) after 7–10 days **(D)** Obverse on Oatmeal Agar (OA) demonstrating compact, darkly pigmented colonies. **(E)** Obverse on Sabouraud Dextrose Agar with chloramphenicol (SDA-C) after 20 days, showing black colonies **(F)** Reverse on SDA-C (representative of all media), highlighting characteristic dark pigmentation. **(G–L)** Microscopic morphology on Lactophenol Cotton Blue preparation. **(G–H)** Creeping, septate, dematiaceous hyphae (white arrows). **(I–L)** Conidiophores arising at right angles from hyphae with sympodial conidiogenesis and denticulate conidiogenous cells (red arrows). Conidia are ellipsoidal with characteristic basal scars (black arrows); green arrows indicate conidiogenous loci. Scale bar **(G–L)** = 20 μm.

Immunosuppression was further tapered and ultimately discontinued. Trimethoprim–sulfamethoxazole was initiated for empiric coverage of toxoplasmosis, given the presence of ring-enhancing CNS lesions in an immunocompromised host. Levetiracetam was concurrently administered for seizure prophylaxis.

On Day 109, the patient was discharged on oral posaconazole (suspension, immediate release) 600 mg daily(45 mg/kg/day) along with trimethoprim–sulfamethoxazole. Despite this prolonged combination antifungal therapy, repeat MRI performed approximately one month after the initial study demonstrated persistence of the dominant lesion with increasing surrounding vasogenic edema and no new intracranial lesions The patient therefore underwent right parietal minicraniotomy with neuronavigation and complete excision of the lesion in an external institution abroad (Day 115). Histopathology demonstrated necrotizing granulomatous inflammation with pigmented septate fungal hyphae, consistent with dematiaceous fungal infection. Fungal cultures grew *R. mackenziei*. Broad fungal PCR was inconclusive, and bacterial PCR detected coagulase-negative staphylococci, which was considered a contaminant in the absence of clinical or microbiological correlation. Postoperatively, the initial posaconazole trough concentration was subtherapeutic (0.17 mg/L), prompting dose escalation and inpatient therapeutic drug monitoring. Following adjustment to 6 mg/kg every 6 hours (oral suspension), the trough concentration increased to 1.1 mg/L. The exact posaconazole formulation used at the external institution was not documented; based on the prescribed regimen, we presume a delayed-release formulation was used, although this could not be confirmed. Postoperative follow-up MRI demonstrated a small persistent enhancing nodule in the right frontal lobe, unchanged from prior imaging, and mild enhancement along the surgical excision tract with a small residual collection, interpreted as postoperative change in the absence of radiologic or clinical evidence of progressive infection. The patient remained clinically stable, afebrile, and neurologically intact on long-term oral posaconazole therapy with therapeutic trough levels and no evidence of progressive disease. The clinical timeline of disease progression and management is illustrated in Fig. 3.

### 2.1 Phenotypic and molecular identification and antifungal susceptibility results

A dematiaceous fungal isolate was received at the UAEU FRL for species-level identification and molecular confirmation. The isolate was assigned an internal accession number and cryopreserved at −80 °C. Phenotypic characterization demonstrated colony and microscopic features consistent with a *Rhinocladiella*-like organism (Fig. 4C–L). Given the morphological overlap among species within the genus, definitive identification required molecular sequencing.

Molecular identification was performed by sequencing the ITS region and the D1/D2 domain of the large-subunit (28S) rRNA gene using previously described primers [7,8]. Species identity was established by BLAST analysis against NCBI GenBank and phylogenetic comparison with reference strains, confirming the isolate as *Rhinocladiella mackenziei*. Sequences were deposited in GenBank under accession numbers [PX777957-ITS] and [PX777966-D1D2].

Antifungal susceptibility testing (AFST) was performed by the Pediatrics and Mycology teams at Christian Medical College, Vellore, India (strain designation: KMT469). The reported minimum inhibitory concentrations (MICs) were as follows: posaconazole, voriconazole, and itraconazole each demonstrated lower MIC of 0.5 μg/mL. In contrast, the isolate showed elevated MICs to amphotericin B (>16 μg/mL), fluconazole (>64 μg/mL), and flucytosine (>64 μg/mL).

## 3 Discussion

### 3.1 Taxonomy, molecular identification, and diagnostic constraints of *R. mackenziei*

Melanized (dematiaceous) fungi are defined by the presence of melanin in their cell walls rather than a single taxonomic lineage [1,2,9]. Within Eurotiomycetes, the order Chaetothyriales, includes several neurotropic opportunistic pathogens with a predilection for CNS infection including *Cladophialophora bantiana*, *Exophiala dermatitidis*, and *R. mackenziei*, all belonging to the Herpotrichiellaceae family and sharing features such as thermotolerance and neurotropism [1,2,[9], [10], [11]].

The genus *Rhinocladiella* comprises approximately 15–20 described species, several of which are recognized human pathogens [12]. These include *R. aquaspersa*, associated with chromoblastomycosis; *R. atrovirens*, implicated in cerebral and subcutaneous phaeohyphomycosis; and *R. basitona*, linked to cutaneous disease, as well as morphologically similar taxa such as *R. similis* [10,13,14]. Given the significant morphological overlap within this genus, molecular identification is essential for accurate species delineation, with ITS sequencing and ribosomal DNA phylogenetic analysis representing the current standard.

### 3.2 Epidemiology and geographic distribution

Since the earliest descriptions of *R. mackenziei* cerebral infection from Saudi Arabia in the 1980s, the majority of cases have originated from the Arabian Peninsula and adjacent arid regions, including the Persian Gulf, Iran, and Pakistan [[3], [4], [5], [6],^15^,^16^]. Early case series consistently demonstrate a strong geographic clustering within these areas, with Saudi Arabia accounting for a substantial proportion of cases [3,4,16]. With the increasing use of molecular diagnostics, additional cases have been recognized outside the Middle East, including Europe and South Asia, challenging earlier assumptions of strict geographic restriction [5,6,10,[17], [18], [19], [20]]. Notably, several European cases occurred in patients of Afghan or Middle Eastern origin, suggesting prior exposure in endemic regions [5,15,16,[18], [19], [20]]. More recently, autochthonous cases reported from Turkey and the Indian subcontinent in patients without travel history raise the possibility of a broader distribution or improved recognition of this pathogen. More broadly, climate change has been proposed as a contributor to the emergence and geographic redistribution of fungal pathogens, although whether it has influenced the epidemiology of *R, mackenziei* remains speculative. [5,15,16,[18], [19], [20], [21]]. Furthermore, molecularly confirmed cases from North Africa, including Morocco, extend the regions potentially at risk for acquisition of this neurotropic fungus [22].

Despite this evolving epidemiological pattern, the environmental reservoir of *R. mackenziei* remains unidentified, as the organism has never been recovered from natural environmental sources to date [3,16,22]. Molecularly confirmed cases of *R. mackenziei* reported since 2010 are summarized in Table 1.

**Table 1:** Molecularly confirmed cases of *Rhinocladiella mackenziei* infection in the post-2010 sequencing era.

Year	Country	Age/Sex	Host/context	Site/specimen	Culture reported	Sequence accession numbers	AFST	Treatment	Outcome	Style ref
2010	India	42 M	Immunocompetent	Solitary cerebral abscess (surgical tissue)	Yes	GQ863214 (ITS)	Not reported	Surgical excision + amphotericin B → itraconazole	Survived	Badali.et al. [4]
2010	France (Afghan native)	80 F	Elderly; colon cancer surgery	Multiple brain abscesses (aspirate)	Yes	HM359013 (ITS); HM359014 (D1/D2)	Not performed (Non-viable culture)	Aspiration + voriconazole → posaconazole	Died (7 mo; non-fungal cause)	Cristini et al. [5]
2010	Qatar	59 F	Breast cancer on chemotherapy	Cerebral abscess (craniotomy + aspiration)	Yes	FJ427211 (ITS); FJ427212 (LSU)	Yes, as per CLSI	L-AmB + VRC → POS	Fatal (64 days)	Taj-Aldeen et al. [3]
2011	Denmark (Somalian native)	50 M	HIV-negative; PCNS lymphoma	Multiple CNS lesions (FFPE biopsy)	No	ITS2 PCR (FFPE); no accessions reported	Not reported	L-AmB + VRC + chemo	Fatal	Pedersen et al. [15]
2011	Pakistan	20–75 y; 5 M/1 F (n = 6)	Diabetes, trauma, postpartum, transplant (mixed)	Cerebral abscesses (tissue/aspirate)	Yes	D1/D2 ± ITS2; no accessions reported	Not reported	Surgery + AmB ± itraconazole	2 alive, 3 died, 1 LTFU (lost to follow up)	Jabeen et al. [6]
2015	Iran	54 M	Immunocompetent	Fronto-parietal brain abscess (tissue-PCR)	No	KJ140287 Biopsy (ITS)	Not performed (no isolate)	AmB + itraconazole	Fatal (2 weeks	Didehdar et al. [10]
2017	United Kingdom	76 M	Immunocompetent	Multiple right parieto-occipital brain abscesses (biopsy + excision)	Yes	Stated as (ITS/18S); no accessions reported	Not reported	Surgical en-bloc excision + voriconazole	Alive (10-month follow-up)	Yusupov et al. [16]
2018	Iran	67 F	Diabetes mellitus; Behçet's disease (on steroids)	Multiple frontal brain abscesses (biopsy + excision)	Yes	MF401515 (ITS)	Yes, as per CLSI	L-AmB + VRC → POS	Fatal (55 days)	Mohammadi et al. [17]
2021	Morocco	60 F	Liver transplant; cirrhosis (HBV/HCV)	Cerebral abscess	Yes	MZ128136 (ITS)	Not reported	AmB-L + 5-FC → VRC → POS; surgery	Fatal	Lafont et al. [22]
2021	Morocco	59 F	Steroids + azathioprine (IBD)	Cerebral abscess + venous thrombosis	Yes	MZ128137 (ITS)	Not reported	VRC + AmB-L + 5-FC → POS; surgery	Survived (26-mo FU)	Lafont et al. [22]
2022	Kuwait	50 F	Kidney transplant on standard immunosuppression	Multiple cerebral + extra-CNS lesions (peri-graft aspirate)	Yes	MZ614624 (ITS)	Not reported	L-AmB + VRC (no surgery)	Fatal (≈2 months)	Al Otaibi et al. [23]
2021	India	65 M	Diabetes mellitus; recent COVID-19 treated with systemic corticosteroids	Multiple cerebral abscesses (surgical tissue, pus)	Yes	Yes (no accessions reported)	Not reported	VRC + AmB-L + 5-FC → POS; surgical excision and drainage	Initial clinical and radiological improvement; deceased at 12 months	Khandhar et al. [19]
2023	Turkey	63 M	Immunocompetent	Frontal brain abscess (aspirate + biopsy)	Yes	OQ804484 (ITS)	Yes, Performed by E-test	AMB → VRC; aspiration + surgical excision	Fatal (3.5 months)	Kucukkaya et al. [18]
2024	India	39 M	Immunocompetent	Left thalamic brain abscess (aspirated pus + excised tissue)	Yes	NR111185 (ITS)	Not reported	Burr-hole aspiration → craniotomy; prolonged VRC	Alive (persistent hemiparesis)	Gupta et al. [20]
2025	UAE *(present BMT pediatric case)*	4 M	BMT/pediatric (UAE)	Osteomyelitis (forearm) and cerebral — clinical isolate	Yes	PX777957-ITS]; PX777966-D1D2	Yes, performed externally; (methodology not specified)	Combined prolonged antifungal therapy Triazoles & AmB ± surgery	Alive	Al Dhaheri et al. (Present)

**Footnote:** AFST, antifungal susceptibility testing; AMB, amphotericin B; BMT, bone marrow transplantation; CAS, caspofungin; CGD, chronic granulomatous disease; CNS, central nervous system; D1/D2, D1/D2 region of the 28S rDNA; FFPE, formalin-fixed paraffin-embedded tissue; FLC, fluconazole; 5-FC, flucytosine; HSCT, hematopoietic stem cell transplantation; ITS, internal transcribed spacer (ITS1–5.8S–ITS2) rDNA region; ITC, itraconazole; MIC, minimum inhibitory concentration (μg/mL); NA, not available; PCR, polymerase chain reaction; POS, posaconazole; RG3, Risk Group 3; SOT, solid organ transplant; VRC, voriconazole; UAE, United Arab Emirates.

### 3.3 Expanding spectrum of neurotropic dematiaceous fungi

Cerebral phaeohyphomycosis is a rare and often fatal infection caused by neurotropic melanized fungi with a predilection for the central nervous system. ESCMID/ECMM guidelines identify *R. mackenziei* and *Cladophialophora bantiana* as the most consistently neurotropic species, whereas *Exophiala dermatitidis* has a broader geographic distribution, particularly in East Asia [24]. The spectrum of recognized causative agents has expanded substantially, now comprising over 150 species across more than 70 genera, largely reflecting advances in molecular diagnostics and taxonomic refinement rather than true increase in incidence [1,[23], [24], [25], [26]]. Additional contributing factors include the growing population of immunocompromised patients, environmental changes, and improved recognition of melanized fungi adapted to arid and hydrocarbon-rich environments [[3], [4], [5], [6],^15^,[23], [24], [25], [26]]. Recent data from Saudi Arabia further illustrate this diversity, with rare causes of cerebral phaeohyphomycosis including *Neoscytalidium dimidiatum*, *Acrophialophora fusispora*, *Chaetomium atrobrunneum*, *Exerohilum rostratum*, and *Fonsecaea pedrosoi*, alongside *C. bantiana* and *R. mackenziei* [27].

### 3.4 Pathogenesis and neurotropism

Melanin in the fungal cell wall is a key virulence factor in dematiaceous fungi, conferring protection against oxidative stress, heat, host enzymes, and immune defenses [2,28]. *R. mackenziei* is classically associated with isolated cerebral infection; however rare cases, including this present case, following traumatic forearm injury in profoundly immunosuppressed patients suggest that peripheral inoculation with subsequent hematogenous spread may be an alternative route to CNS disease [[3], [4], [5], [6],^10^,^17^]. Prolonged latency has been proposed, exemplified by an Afghan-born patient in whom cerebral disease manifested nearly two decades after last exposure to an endemic region, raising the possibility that dormant infection may persist for years prior to clinical presentation [5]. At a molecular level, genomic and transcriptomic analyses of black yeasts within *Chaetothyriales* have identified enrichment of gene families involved in melanin biosynthesis, cytochrome P450–mediated metabolism of aromatic and hydrocarbon substrates, iron acquisition, and multiple stress-response pathways, which likely may contribute to environmental persistence and neurotropic potential [[29], [30], [31]].

### 3.5 Host factors and transplantation-related risk in the context of HSCT

In CGD, defects in the phagocyte NADPH oxidase complex impair intracellular killing of catalase-positive organisms and fungi, predisposing patients to persistent infections and granulomatous inflammation [32]. Although allogeneic HSCT restores immune function, it introduces a period of heightened vulnerability due to conditioning regimens, delayed immune reconstitution, and ongoing immunosuppression. As a result, HSCT recipients remain at increased risk for invasive fungal infections (IFI) [25,33]. A recent meta-analysis identified haploidentical transplantation, GVHD of grade ≥2, and systemic corticosteroid exposure as key risk factors for IFI in this population, reflecting prolonged impairment of T-cell–mediated antifungal immunity [33]. The present case exemplifies this convergence of risk factors, with *R. mackenziei* osteomyelitis following minor trauma during intensified immunosuppression, which suggests peripheral inoculation as a potential extracerebral portal of entry.

### 3.6 Antifungal susceptibility and therapeutic considerations

In HSCT recipients, particularly those with GVHD, mold active triazoles such as posaconazole is preferred, with voriconazole as an alternative, given broader mold coverage and more favorable toxicity profile [34]. As summarized in Table 2, *R. mackenziei* demonstrates reduced in vitro susceptibility to amphotericin B, with consistently elevated MICs across reference strains and clinical isolates. In contrast, triazoles, particularly posaconazole and itraconazole, generally demonstrate lower MICs, while voriconazole activity remains more variable [3,17,18,35]. In the absence of established clinical breakpoints, MIC values must be interpreted descriptively. These patterns highlight the risk of inappropriate antifungal selection in the absence of molecular identification and susceptibility testing.

**Table 2:** Comparative antifungal susceptibility data for *R. mackenziei* isolates reported in published studies and the present case.

Year	Country	Method	AMB MIC (μg/mL)	ITC MIC (μg/mL)	VRC MIC (μg/mL)	POS MIC (μg/mL)	Other (μg/mL)	References
2010	Reference strains (*n* = 10) performed in CBS-KNAW, The Netherlands	CLSI M38	2–16	0.063–0.25	0.25–2	0.016–0.063	FLC 16–64; ISA 0.25–1; CAS (MEC) 1–8; ANID (MEC) 1–8	Badali et al. [35]
2010	Qatar	CLSI M38	8–16	≤0.03	≤0.03	≤0.03	ANID MEC 2–4; CAS & MICA (MEC) 1	Taj-Aldeen et al. [3]
2018	Iran	CLSI M38	16	0.125	0.25	0.063	LANO/LULI 0.002	Mohammad et al. [17]
2023	Turkey	E-test	>32	0.25	0.032	0.064	CAS 0.002; ANID <0.002	Kucukkaya et al. [18]
2025	UAE (present case- performed in India)	Not specified	>16	0.5	0.5	0.5	FLC >64; 5-FC >64	Present Study

**Footnote:** AFST, antifungal susceptibility testing; MIC, minimum inhibitory concentration (μg/mL); MEC, minimum effective concentration; CLSI, Clinical and Laboratory Standards Institute; AMB, amphotericin B; ITC, itraconazole; VRC, voriconazole; POS, posaconazole; FLC, fluconazole; 5-FC, flucytosine; CAS, caspofungin; MICA, micafungin; ANID, anidulafungin; LANO, lanoconazole; LULI, luliconazole.

No established clinical breakpoints exist for *R. mackenziei*; MIC values are reported for descriptive comparison only.

Current ESCMID/ECMM guidance emphasizes complete surgical excision of cerebral lesions as the intervention most strongly associated with improved outcomes, while amphotericin B–based therapy alone is generally associated with poor response. Triazoles, particularly voriconazole and posaconazole, have shown benefit in selected cases, and combination therapy may be considered when surgery is not feasible [24]. In the present case, persistent disease despite combination antifungal therapy prompted complete surgical excision, followed by long-term posaconazole with sustained clinical stability, aligning with published susceptibility data and guideline recommendations.

## 4 Conclusion

Our case is unique in its pediatric presentation, extracerebral osteomyelitic entry site, post-HSCT setting, and favorable outcome following combined antifungal and surgical management. It underscores the need for early imaging, molecular diagnosis, and aggressive multidisciplinary care when managing suspected dematiaceous fungal infections of the CNS.

## Data availability

All generated sequences have been deposited in the NCBI GenBank database under accession numbers [PX777957-ITS] and [PX777966-D1D2].

## Funding

This study was supported by a United Arab Emirates University Start-up Grant (Grant No. G00004532) and the UAE–NIH Collaborative Award (Research Grant Reference No. AJF-NIH-13-UAEU), both awarded to Fatima Al Dhaheri.

## CRediT authorship contribution statement

**Tasneem Shamsi Basha:** Conceptualization, Data curation, Investigation, Writing – original draft. **Hari Pankaj Vanam:** Data curation, Formal analysis, Investigation, Methodology, Writing – original draft, Writing – review & editing. **Akela Ghazawi:** Formal analysis, Methodology, Visualization, Writing – review & editing. **Mansi Sachdev:** Data curation, Investigation, Writing – review & editing. **Shazia Chiken:** Data curation, Investigation, Writing – review & editing. **Rubina Monga:** Data curation, Investigation, Writing – review & editing. **Fatima Al Dhaheri:** Conceptualization, Funding acquisition, Investigation, Methodology, Project administration, Supervision, Writing – review & editing.

## Conflict of interest

The authors declare no conflicts of interest.
